# Supplementary material for: High spatial dynamics-photoluminescence imaging reveals the metallurgy of the earliest lost-wax cast object
Source: Nat Commun. 2016 Nov 15;7:13356. doi: 10.1038/ncomms13356 (PMC5116070; doi:10.1038/ncomms13356)
Supplement: Supplementary Information — Supplementary Figures 1-10, Supplementary Note 1, Supplementary Methods and Supplementary References [file ncomms13356-s1.pdf]

Supplementary Information

Supplementary Figures:

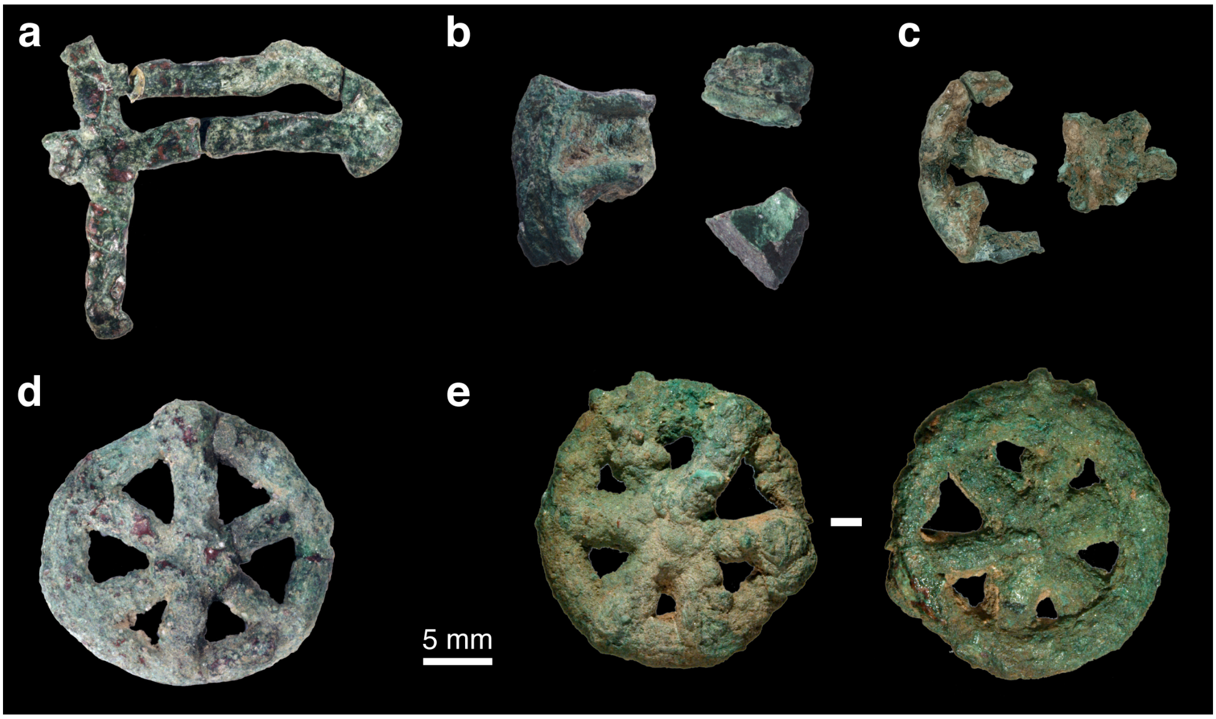

Supplementary Figure 1 | Lost-wax cast ornaments discovered during excavation of the MR2 site at Mehrgarh (Early Chalcolithic, end of period III, 4500–3600 BC). (**a**) Copper-based fragmentary ornament (inv. n° MR.81.2X.49.01) discovered during the 1980–1981 excavation of a group of mud brick buildings for storage (MR2, sector X). The metal fragment was found outside of the buildings in a black sediment layer that also contained many potsherds, flints and animal bones (square X5B, see Jarrige *et al.* 1995 fig 7.12 p. 341[1]). This layer corresponds to the second and oldest excavated level, but is near the present surface because of erosion of the building, © C2RMF, D. Bagault. (**b**) Three fragments probably from a single ornament (inv. n° MR.81.2X.106.01, MR.81.2X.106.02, MR.81.2X.106.03) discovered during the 1980–1981 excavation of sector X. Like **a** found in the second and oldest excavated level and outside of the buildings, these metal fragments were in a soil mixed with numerous mud brick remains (X9C square, see Jarrige *et al.* 1995 fig 7.12 p. 341[2]), © C2RMF, D. Bagault. (**c**) Fragmentary wheel-shaped ornament (inv. n° MR.83.02.05.01) discovered during the 1982–1983 excavation of a 85 m² graveyard located near the buildings of sector X. A unique level of graves was discovered just below the present surface (73 skeletons). The wheel-shaped ornament was discovered close to the skull of a woman buried in the individual tomb H 33 (square R2J, see Sellier & Samzun 1983 fig. 3 p. 74[2]). © C2RMF, B. Mille. (**d**) Wheel-shaped ornament (inv. n° MR.85.03.00.01) discovered by Anaick Samzun in 1985 from a surface recollection at the MR2 site. Despite the fact that the copper artefact was not discovered in its primary position, it belongs to the Early Chalcolithic period since the MR2 site dates entirely from this period. This artefact is the one discussed in detail in this paper, © C2RMF, D. Bagault. (**e**) Wheel-shaped ornament (inv. n° MR.90.02.00.01) discovered in 1990 during a late surface recollection of the Chalcolithic site MR2 (left: front side, right: back side), © C2RMF, B. Mille.

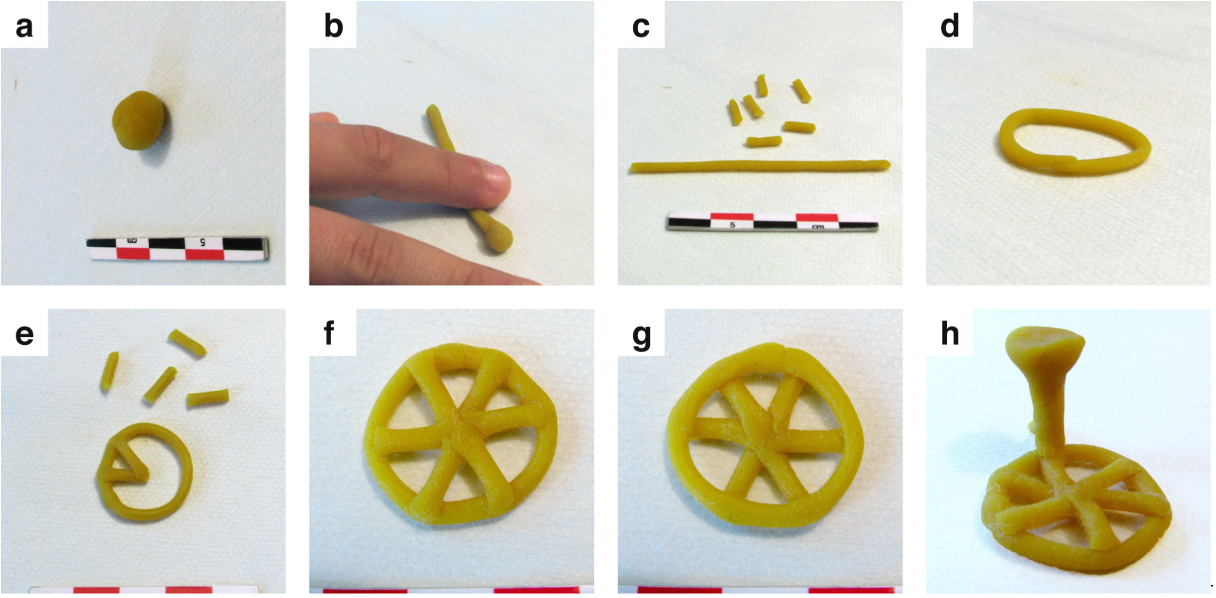

Supplementary Figure 2 | Manufacturing of the wax model of the amulet from Mehrgarh by the lost-wax casting process. Based on visual examination of the wheel-shaped amulets from Mehrgarh (Fig. S1 **c**–**e**), the following *chaîne opératoire* is proposed:

– a model was first shaped by manufacturing small rods in a very ductile material that melts at low temperature such as beeswax (**a**–**c**),
– each wax piece was welded to the other by a slight heating of their extremities, a pouring basin and a sprue were added (**e**–**h**),
– the wax model was invested by a clay mixture to form a mould,
– the mould was heated upside down to run out the wax, baking was extended at higher temperature to harden the mould and drive out any moisture,
– copper was poured in the mould, taking place of the wax,
– after cooling, the mould was broken and the casting was finished by cold working such as cutting the sprue and polishing.

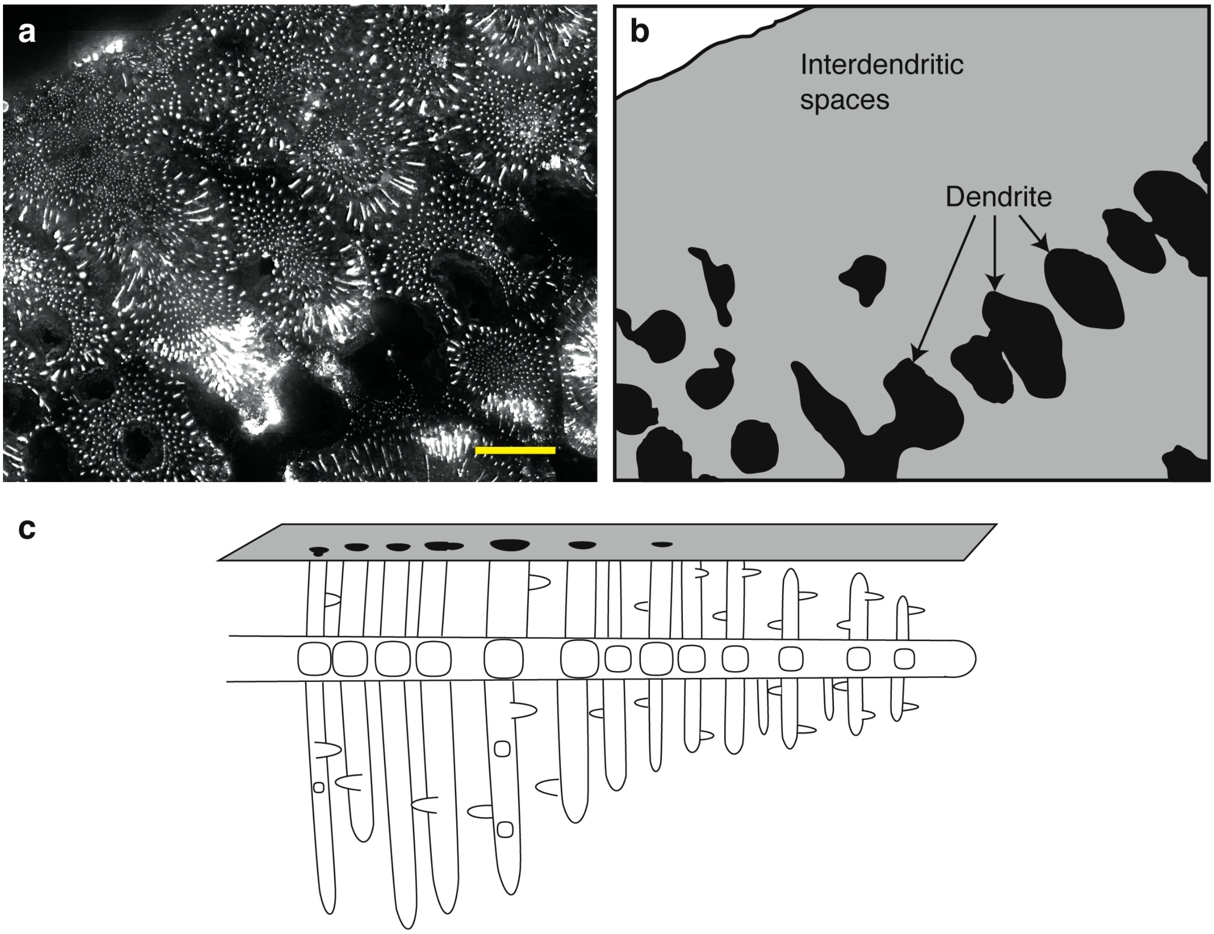

Supplementary Figure 3 | Metallographic dendritic structure with large interdendritic spaces. (**a**) PL image of the wheel under 420–480 nm excitation and 850–1020 nm band-pass emission. Scale bar: 100 μm. (**b**) Schematic representation of the dendritic and interdendritic structures from the PL image in **a**. (**c**) 3D schematic representation of the equatorial cross-section represented in **b** cutting through a dendrite.

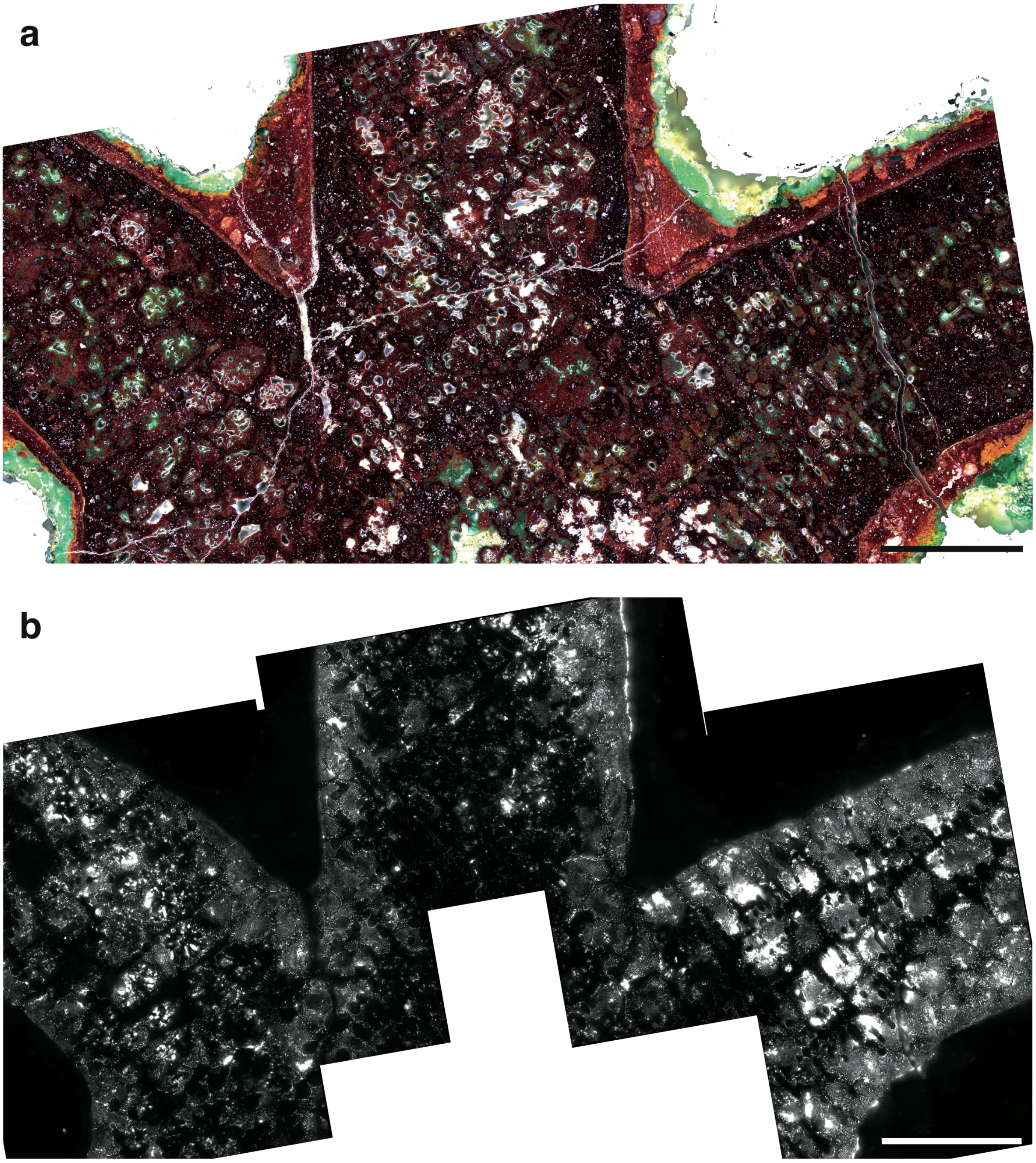

Supplementary Figure 4 | Homogeneity of the eutectic microstructure over the whole wheel-shaped artefact. (**a**) Dark-field microscopy image of three spokes of the amulet. (**b**) PL images of the same three spokes under 420–480 nm excitation illumination and 850–1020 nm band-pass emission (40× objective, N.A.=0.6). The regular PL emission of the micrometric-size rod patterned phase is observed over millimetres in the interdendritic spaces. Scale bars: 1 mm.

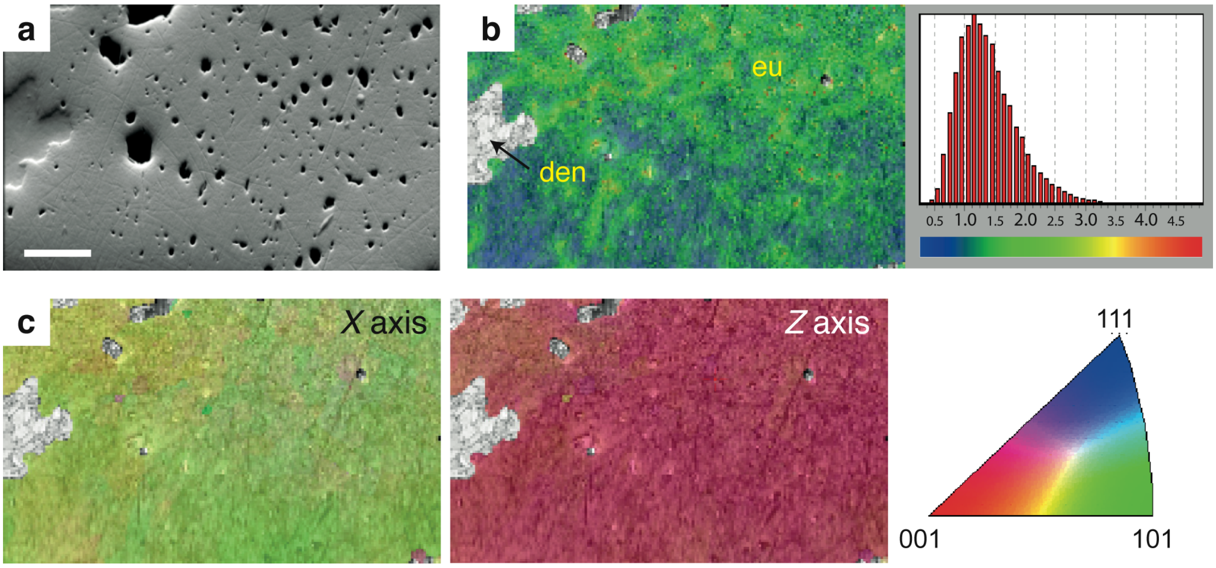

Supplementary Figure 5 I EBSD maps showing the excellent agreement between eu-Cu_2O and co-Cu_2O crystallographic orientations. (**a**) Topographic backscattered electron image (forward scatter detector FSD, 4 quadrants). (**b**) EBSD misorientation angle map and histogram for the Cu_2O phase **eu**. The local misorientation is lower than 3.5°, corresponding to a very limited local misorientation between pixels. It is calculated for each pixel by difference between the mean value from 8 neighbouring pixels. Pixel step=744 nm. Clinoatacamite (in grey) is identified in the dendrite **den** and Cu_2O in the interdendritic space **eu**. (**c**) Inverse pole figure (IPF) for the *X* and *Z* axes (left and centre, resp.), and corresponding IPF colouring (right), revealing a very homogeneous crystallographic distribution within the Cu_2O-containing interdendritic space. Scale bar for all images: 20 μm.

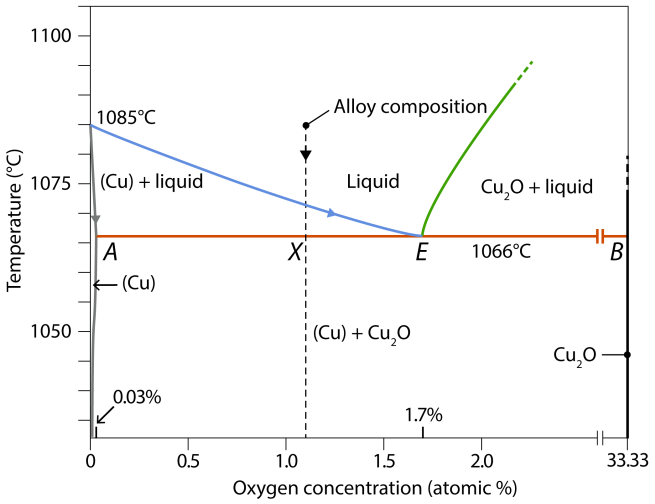

Supplementary Figure 6 l Enlarged view of the Cu–O phase diagram in the Cu–Cu_2O eutectic region. From the initial composition of the melt alloy, the system followed: solidification of Cu dendrites from liquid **a**, solidification of the Cu–Cu_2O eutectic at T=1066°C **b**, and final composition at the solid state of the artefact below 1066°C **c**. E: eutectic solidification point at 1.1 at% of oxygen. Adapted from Neumann, J. P., Zhong, T., & Chang, Y. A. The Cu–O (Copper-Oxygen) system. *Journal of Phase Equilibria* **5,** 136–140 (1984).

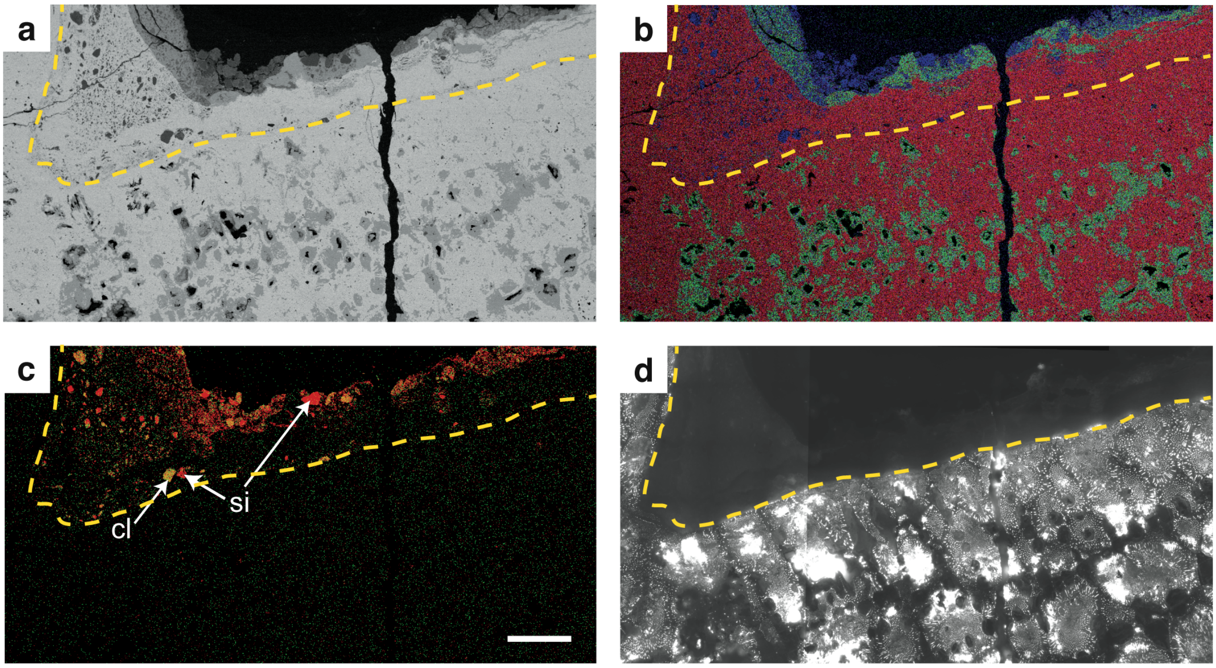

Supplementary Figure 7 | Limit of the original surface of the artefact, delineated by soil particles within the external corrosion layer and eutectic PL emission. (**a**) Ghost metallic structure with dendrites and homogeneous interdendritic spaces from SEM-BEI at 10 kV. The yellow dotted line indicates the original surface. The original surface precisely delimits the initial shape of the artefact. Black: resin, dark grey: soil species, grey: Cu(**II**) compounds, white grey: Cu_2O. (**b**) RGB false colour image of Cu (red), Cl (green), O (blue) from EDS maps. (**c**) False colour image of Si (red) and Al (green) from EDS X-ray maps. The maps reveal the presence of elements typically found in soils that are embedded in an external cuprous oxide layer. **cl**: Si–Al–O compounds (clay), **si**: Si–O compounds (SiO_2). (**d**) Photoluminescence image at 850–1020 nm emission under 420–480 nm excitation. The luminescent rod-like microstructures delineate the original surface of the artefact. Scale bar for all figures: 300 μm.

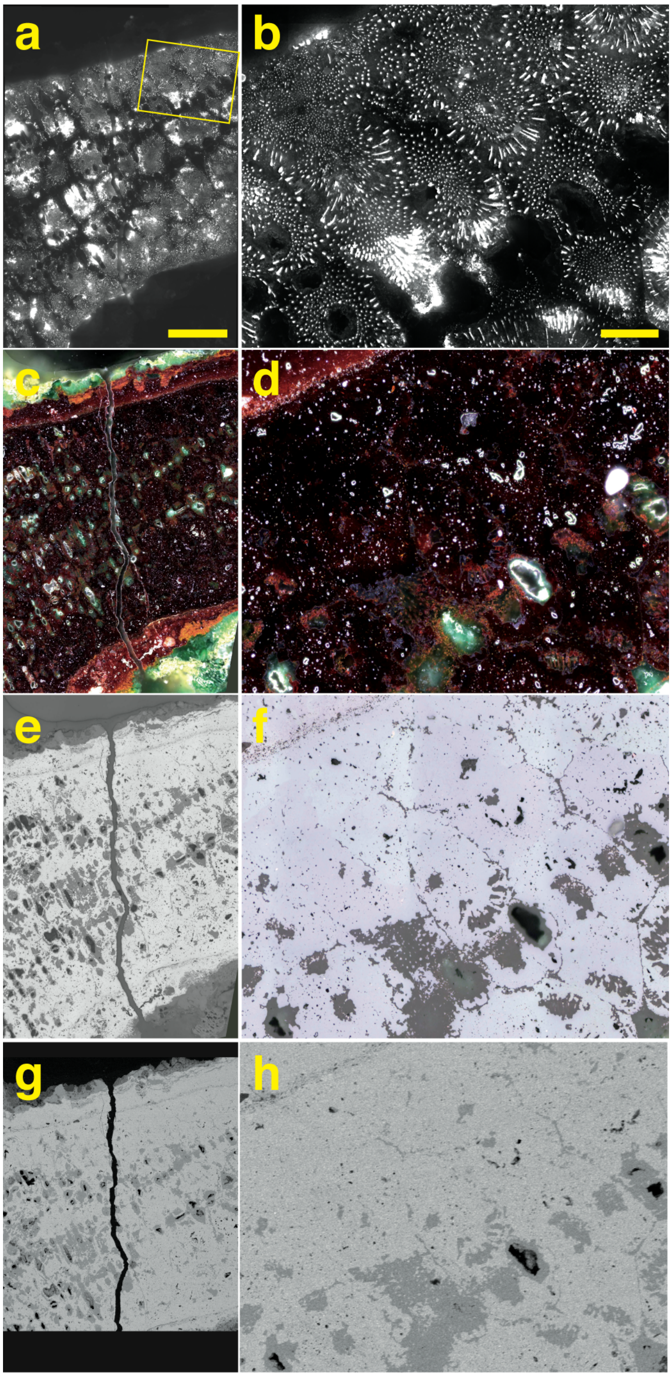

Supplementary Figure 8 | Comparison of light microscopy, DUV photoluminescence and electron microscopy images. PL images (**a**) and close-up view (**b**) corresponding to the area delimited by the yellow rectangle in **a** of the amulet (420–480 nm excitation, 850–1020 nm band-pass emission). 40× objective, N.A.=0.6 (left), and 100× objective, N.A.=1.25 (right). (**c**,**d**) Dark-field light microscopy images of the same areas as in **a** and **b**, respectively. (**e**,**f**) Bright-field light microscopy images of the same areas as in **a** and **b**, respectively. (**g**,**h**) Electron microscopy images (SEM-BEI, 10 kV) of the same areas as in **a** and **b**, respectively. Scale bars: 500 μm (left), and 100 μm (right).

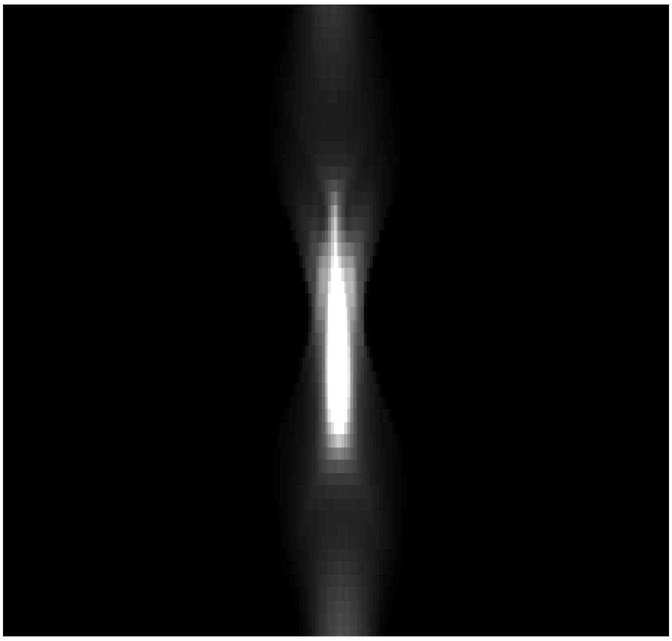

Supplementary Figure 9 | Experimental optical point spread function (PSF) of the micro-imaging set up. The PSF was generated from a stack of 117 images collected with a 650 nm-step in *z*, using excitation at 385 nm and 850–1020 nm detection range collected on a 400-nm diameter CdS crystal in an immersion medium (Immersol, Zeiss). This experimental PSF was generated using the PSF Distiller Tool (Huygens software, Scientific Volume Imaging).

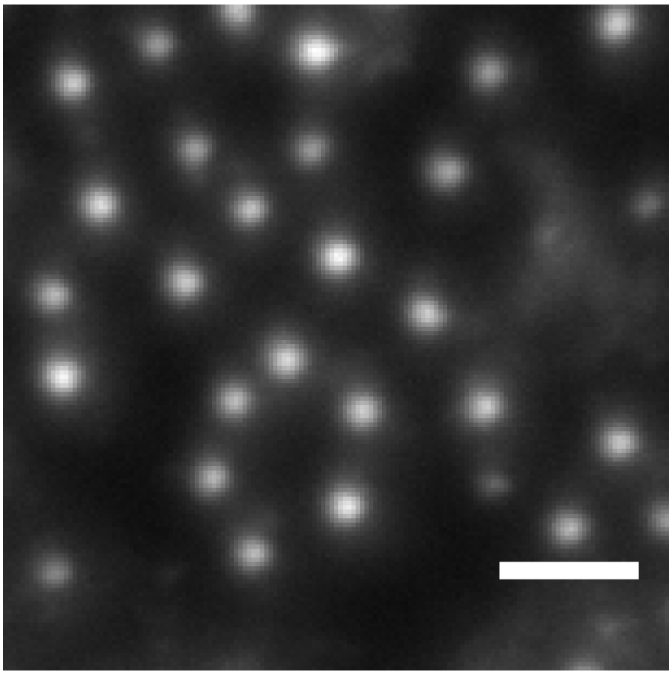

Supplementary Figure 10 | Microstructure of the eutectic pattern. Photoluminescence images of an area of the spokes under 420–480 nm excitation and 850–1020 nm band-pass emission (100× objective, N.A.=1.25). The regular PL emission pattern at micrometric length scale is observed within the interdendritic spaces, and demonstrates the interest of the ca. 1-μm lateral resolution. Scale bar: 5 μm.

Supplementary Note 1. Determination of the oxygen content in the original cast amulet.

The oxygen content (X) in the original copper melt can be determined using the Cu–O equilibrium phase diagram (Supplementary Fig. 6). In the solid state, the hypo-eutectic structure is composed of Cu (dendrites) and eutectic $Cu–Cu_2O$ (interdendritic spaces). Thus the amount of oxygen (X) is directly related to the proportion of the eutectic in the alloy.

Applying the level rule[3] at the eutectic transformation (1066°C), the weight ratio R_{wt} between the dendrites and the eutectic proportions (W) can be determined from:

$$(1) \qquad R_{\mathrm{wt}} = \frac{W(\mathrm{dendrite})}{W(\mathrm{eutectic})} = \frac{E-X}{X-A}$$

with A = 0.0076 wt% (~0.03 at%) and E = 0.434 wt% (~1.7 at%), the oxygen contents at the maximum solubility of the copper phase and of the eutectic respectively.

Thus, the oxygen concentration (X) of the original cast object is defined by:

$$(2) \qquad X = \frac{E + R_{\mathrm{wt}} \times A}{1 + R_{\mathrm{wt}}}$$

The weight ratio R_{wt} can be defined from the volume proportion R_{v} between the dendrites and the eutectic as:

$$(3) \qquad R_{\mathrm{wt}} = R_{\mathrm{v}} \times \frac{\rho_{\mathrm{eutectic}}}{\rho_{\mathrm{Cu}}} \approx R_{\mathrm{v}}$$

considering the density values ρ_{Cu} and ρ_{eutectic} of respectively 8.95 and 8.79 g cm^{-3}*.

From the whole amulet cross-section examination of the surface, the distribution of the phases is relatively uniform. We can thus assume a homogeneous distribution of the phases within the volume, as in a cubic system the surface ratio R_s of the different phases can be estimated equal to their volume ratio R_v** [3]. In this context:

$$(4) \qquad R_{\mathrm{v}} \approx R_{\mathrm{s}}$$

From BEI and PL measurements of the metallurgical ghost structure, the area of eutectic on the whole amulet is found equal to 66 ± 10 % of the total surface.

Then:

$$(4) \qquad R_{\mathrm{v}} \approx R_{\mathrm{s}} = \frac{34}{66} = 0.55 \pm 0.23$$

Applying (4) in (3), then (2) gives:

$$(5)\ X \sim \frac{(0.434 + 0.52 \times 0.0076)}{1 + 0.52} = 0.29 \pm 0.05 \text{ wt\%}$$

Consequently, the total oxygen content of the original alloy composition is ***X* ~ 0.29 ± 0.04 wt% (~ 1.1 at%)**, in good agreement with an expected hypoeutectic composition.

*** Determination of the eutectic density $\rho_{eutectic}$.** The total volume of the eutectic is equal to the sum of the volume of the eu-Cu and eu-Cu_2O phases:

$$V_{\text{total}}(\text{eutectic}) = V(\text{eu-Cu}) + V(\text{eu-Cu}_2\text{O})$$

Thus, the weight fraction (*W*) of each phase and their density are related as:

$$\frac{1}{\rho_{\text{eutectic}}} = \frac{W(\text{eu-Cu})}{\rho_{\text{Cu}}} + \frac{W(\text{eu-Cu}_2\text{O})}{\rho_{\text{Cu}_2\text{O}}}$$

Applying the lever rule at the point E, the weight fraction of each phase is then:

$$\frac{1}{\rho_{\text{eutectic}}} = \frac{\frac{(B-E)}{\rho_{\text{Cu}}} + \frac{(E-A)}{\rho_{\text{Cu}_2\text{O}}}}{(B-A)}$$

Thus:

$$\rho_{\text{eutectic}} = \frac{(11.18 - 0.0076)}{\left[\frac{(11.18 - 0.434)}{8.95} + \frac{(0.434 - 0.0076)}{6.11}\right]} = 8.79 \text{ g cm}^{-3}$$

**** Determination of the theoretical volume ratio R_v and experimental surface ratio R_s within the eutectic Cu–Cu_2O.** The assertion of a homogeneous spatial distribution of the phases is here validated from comparison between the theoretical volume ratio (R_v) and the experimental surface ratio (R_s) between Cu_2O and Cu in the eutectic.
R_v is estimated as follows:

$$R_v(\text{eutectic}) = \frac{V(\text{eu-Cu}_2\text{O})}{V(\text{eu-Cu})} = \frac{W(\text{eu-Cu}_2\text{O})}{W(\text{eu-Cu})} \times \frac{\rho_{\text{Cu}}}{\rho_{\text{Cu}_2\text{O}}} = \frac{E-A}{B-E} \times \frac{\rho_{\text{Cu}}}{\rho_{\text{Cu}_2\text{O}}} = \frac{0.434 - 0.0076}{11.18 - 0.434} \times \frac{8.95}{6.11} = 0.058$$

Within the eutectic, the experimental value of R_s(eutectic) = 0.069 (± 0.019) is determined from the PL image analysis of the amulet cross-section on 6 eutectic areas from 0.01 to 0.05 mm^2. It is here determined from the ratio of (eu-Cu_2O) to (co-Cu_2O) areas. Luminescent eu-Cu_2O areas are measured at 850–1020 nm with under excitation at 420–480 nm.

In the eutectic, a good agreement between the *R*v (0.058) and *R*s (0.069 ± 0.019) ratios is thus found. This confirms that the surface ratio R_s of the eutectic versus dendrite phases can be compared to the volume ratio R_v as done in equation (3).

Supplementary References

1. Jarrige, C., Jarrige, J.-F., Meadow, R. H., Quivron, G. *Mehrgarh Field Reports 1974-1985, From Neolithic Times to the Indus Civilization* (Karachi: The Department of Culture and Tourism,

Government of Sindh in collaboration with the French Ministry of Foreign Affairs, 1995).
2. Samzun, A., Sellier, P. Découverte d'une nécropole Chalcolithique à Mehrgarh, Pakistan. *Paléorient* **9**, 69–79 (1983).
3. Baker, H. Introduction to Alloy Phase Diagrams, in *Alloy Phase Diagrams, ASM Handbook Vol. 3* (ASM International, 2004).

Supplementary Methods:

Supplementary Method 1 | Characteristics of the amulet from Mehrgarh. The ornament with inventory number MR.85.03.00.01 could be studied in detail (Fig. 1C–D). A visual inspection indicated that its 'spoked wheel' shape consists of six small rods lying on a ring of 20-mm diameter. At the centre of the wheel, the spokes were clearly pressed on each other until a junction was obtained by superposition; the base of each spoke was attached to the support-ring using the same technique. Both the spokes and the support ring are circular in section. Only a wax-type material, *i.e.* easily malleable and fusible, could have been used to build the corresponding models. This wheel-shaped amulet cannot result from casting in a permanent mould: this shape could not have been withdrawn without breaking the mould, as no plane intercepts jointly the equatorial symmetry planes of the support ring and of the spokes without inducing an undercut. The artifact was therefore cast using a lost-wax process (Supplementary Fig. 2).

A first campaign of measurements was performed ten years ago but the wheel-shaped amulet could only be exhaustively described through novel advanced imaging. X-ray radiographs showed that it is corroded from its surface to its core. SEM examination of the equatorial section of the amulet corroborated the complete corrosion of the artifact, yet showed locally a fossilized dendritic structure, confirming a casting process. X-ray microanalyses on small areas highlighted Cu, O and Cl in the dendrites and Cu and O in the interdendritic space. Raman spectra allowed identifying the corrosion compounds: clinoatacamite $Cu_2(OH)_3Cl$ in the dendrite and cuprous oxide Cu_2O in the interdendritic space. However, corrosion precluded any further understanding of the manufacturing and metallurgical processes.
